# Drug repurposing strategies of relevance for Parkinson’s disease

**DOI:** 10.1002/prp2.841

**Published:** 2021-07-26

**Authors:** Edward J. R. Fletcher, Thomas Kaminski, Gareth Williams, Susan Duty

**Affiliations:** ^1^ King’s College London Institute of Psychiatry, Psychology & Neuroscience Wolfson Centre for Age‐Related Diseases London UK

**Keywords:** clinical observation, epidemiological correlation, genome‐wide association studies, target identification, transcriptional profiling

## Abstract

Parkinson's disease is a highly disabling, progressive neurodegenerative disease that manifests as a mix of motor and non‐motor signs. Although we are equipped with some symptomatic treatments, especially for the motor signs of the disease, there are still no established disease‐modifying drugs so the disease progresses unchecked. Standard drug discovery programs for disease‐modifying therapies have provided key insights into the pathogenesis of Parkinson's disease but, of the many positive candidates identified in pre‐clinical studies, none has yet translated into a successful clinically efficacious drug. Given the huge cost of drug discovery programs, it is not surprising that much attention has turned toward repurposing strategies. The trialing of an established therapeutic has the advantage of bypassing the need for preclinical safety testing and formulation optimization, thereby cutting both time and costs involved in getting a treatment to the clinic. Additional reduced failure rates for repurposed drugs are also a potential bonus. Many different strategies for drug repurposing are open to researchers in the Parkinson's disease field. Some of these have already proven effective in identifying suitable drugs for clinical trials, lending support to such approaches. In this review, we present a summary of the different strategies for drug repurposing, from large‐scale epidemiological correlation analysis through to single‐gene transcriptional approaches. We provide examples of past or ongoing studies adopting each strategy, where these exist. For strategies that have yet to be applied to Parkinson's disease, their utility is illustrated using examples taken from other disorders.

## Graphical abstract

The journey of exenatide shows the potential that repurposing strategies based upon initial epidemiological observations offer for identifying new disease‐modifying therapies for Parkinson's disease.

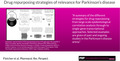

**Abbreviations**
17‐DMAG: 17‐dimethylaminoethylamino‐17‐demethoxygeldanamycin
6‐OHDA: 6‐hydroxydopamine
ABL1: abelson tyrosine protein kinase 1
AD: Alzheimer's disease
CMAP: connectivity mapping
eQTL: expression qualitative trait loci
FGF20: fibroblast growth factor 20
GLP‐1: glucagon‐like peptide‐1
GTEx: Genotype‐Tissue Expression
GWAS: genome wide association studies
iPSC: induced pluripotent stem cell
MPTP: 1‐methyl‐4‐phenyl‐1,2,3,6‐tetrahydropyridine
PD: Parkinson's disease
T2DM: type II diabetes mellitus

## 1 INTRODUCTION

Parkinson's disease (PD) is the second most prevalent age‐related neurodegenerative disease. Typical pathology of PD involves the degeneration of dopaminergic nigrostriatal neurones which is largely responsible for the motor symptoms, while dysfunction in other brain regions contributes to development of the non‐motor symptoms such as anxiety and cognitive disturbances. Despite the number of available PD therapies, including dopamine agonists and the dopamine precursor, levodopa, none slow or reverse disease progression and this raises the first of two motivating factors in the drive for new therapeutics. The second motivating factor is that the number of PD cases is projected to rise due to an ever aging population and the downward trend in smoking, two well‐known effectors of PD risk.^1^ Indeed PD is the fastest growing neurological disorder globally. In 2016, a reported 6.1 million people worldwide were living with PD, reflecting a greater than 20% increase in age‐standardized prevalence rates between 1990 and 2016.^2^ Unfortunately, the slow rate of disease progression characteristic of PD makes the design of clinical trials for potential disease‐modifying therapies problematic as these are powered by the drug's effect on slowing decline and thus require either large cohort sizes or long treatment regimes.

In light of the length and leakiness of the traditional drug development pipeline, researchers have increasingly turned toward harnessing the potential of existing therapeutics through drug repurposing. This application of established therapeutics to indications for which they were not originally prescribed has the advantage of bypassing the protracted and costly preclinical safety testing and optimization in drug development. Additionally, marketed drug prescription records can inform the prioritization of repurposing candidates. Alternatively, in the case of natural compounds, low‐cost trials can be designed based on the self‐administration of the drug in the form of a food supplement.

The antiviral agent, amantadine, is the earliest example of a repurposing success in PD, long before “repurposing” became established. Following observation in the early 1960s of a patient experiencing relief of motor symptoms when taking the medication for influenza, amantadine has become part of the standard PD treatment protocol for early motor symptoms and especially for tackling levodopa‐induced dyskinesia.^3^ Although other examples of drugs repurposed in this way exist, they are the exception. A more hypothesis‐driven approach for repurposing is through the emergence of a new target for intervention and in this case an off‐the‐shelf approach can be adopted based on the extensive publicly available drug interaction data. However, repurposing has the additional potential to harness the complex pharmacology of some medications, where there are either multiple or ill‐defined modes of action, in diseases with no clearly defined target for pharmacological intervention. The richness of high content quantitative data on drug activities and disease states has opened up the possibility of discovering drug candidates based on their reversing disease associated phenotype changes. In addition, databases containing information on existing drugs, such as protein–protein interaction, activity profiles across cell panels, and gene expression profiles can be interrogated to identify potential drugs for repurposing to suit a specific hypothesis. Finally, hypotheses for the repurposing of drugs can be generated based on the interrogation of epidemiological data such as prescription and other healthcare records. In these ways, mechanisms to help accelerate drug discovery can be open to academic laboratories.

In this review, we provide an overview of the different routes that drug repurposing can follow, with specific emphasis on PD. The discussion ranges from epidemiological, observational studies through to single‐gene transcriptional approaches. Selected examples are given of past and ongoing studies in the PD arena. Strategies with therapeutic potential, but not yet applied to PD, are discussed in the context of other diseases.

## 2 CURRENT AND POTENTIAL REPURPOSING STRATEGIES IN PARKINSON’S DISEASE

### 2.1 Isolated observations in patients

One of the more unusual routes to repurpose drugs for PD is based on the serendipitous reporting of improved symptoms, by PD patients or their healthcare team, associated with medications taken for comorbidities. The case of amantadine, recognized when given to a patient with influenza, was already highlighted in the Introduction. Amantadine is now used worldwide to help control the dyskinetic side effects that emerge in some people following long‐term levodopa administration. In this case, the pharmacology behind the beneficial anti‐dyskinetic effect of amantadine, that of NMDA receptor antagonism^4^ or, as more recently proposed, blockade of striatal inwardly rectifying K^+^ channel K _ir_ 2.1,^5^ bears no resemblance to its original antiviral action. Similarly, the anticonvulsant zonisamide, also identified through clinical observation in a single patient to provide relief against levodopa‐induced dyskinesia and now approved for such use in Japan,^6^ is believed to produce its beneficial effects in PD through different pharmacological actions than those underlying its anticonvulsant activity.^7^ More often, drugs are identified as potential repurposing candidates through more extensive observational studies in which case the pharmacological target proposed to bring relief in PD is often the same as that mediating efficacy in the original indication, as exemplified below.

### 2.2 Observational studies

Positioned at one extreme of the repurposing spectrum lies epidemiological correlation analysis. This approach is increasingly viable due to the availability, albeit restricted, of detailed medical records such as prescription data, from which disease incidence in cohorts prescribed a particular drug can be compared to non‐users. The longitudinal nature of these observational studies means they are also much more likely to identify drugs which have disease‐modifying potential. However, it cannot be assumed that all drugs that reduce the incidence of PD will act to slow down progression of established disease. A good example of this is the recognized inverse association between caffeine consumption and the risk of developing PD^8^ which has not resulted in the effective use of caffeine as a disease modifying treatment. While a wealth of support for the neuroprotective effects of caffeine was generated in preclinical animal models of PD (reviewed recently^9^), the latest Café‐PD trial, a multicenter double‐blind, randomized, placebo‐controlled phase 2 trial found no improvement in motor measures with caffeine compared to placebo, and indeed revealed a worsening of cognitive signs.^10^

**FIGURE 1:**
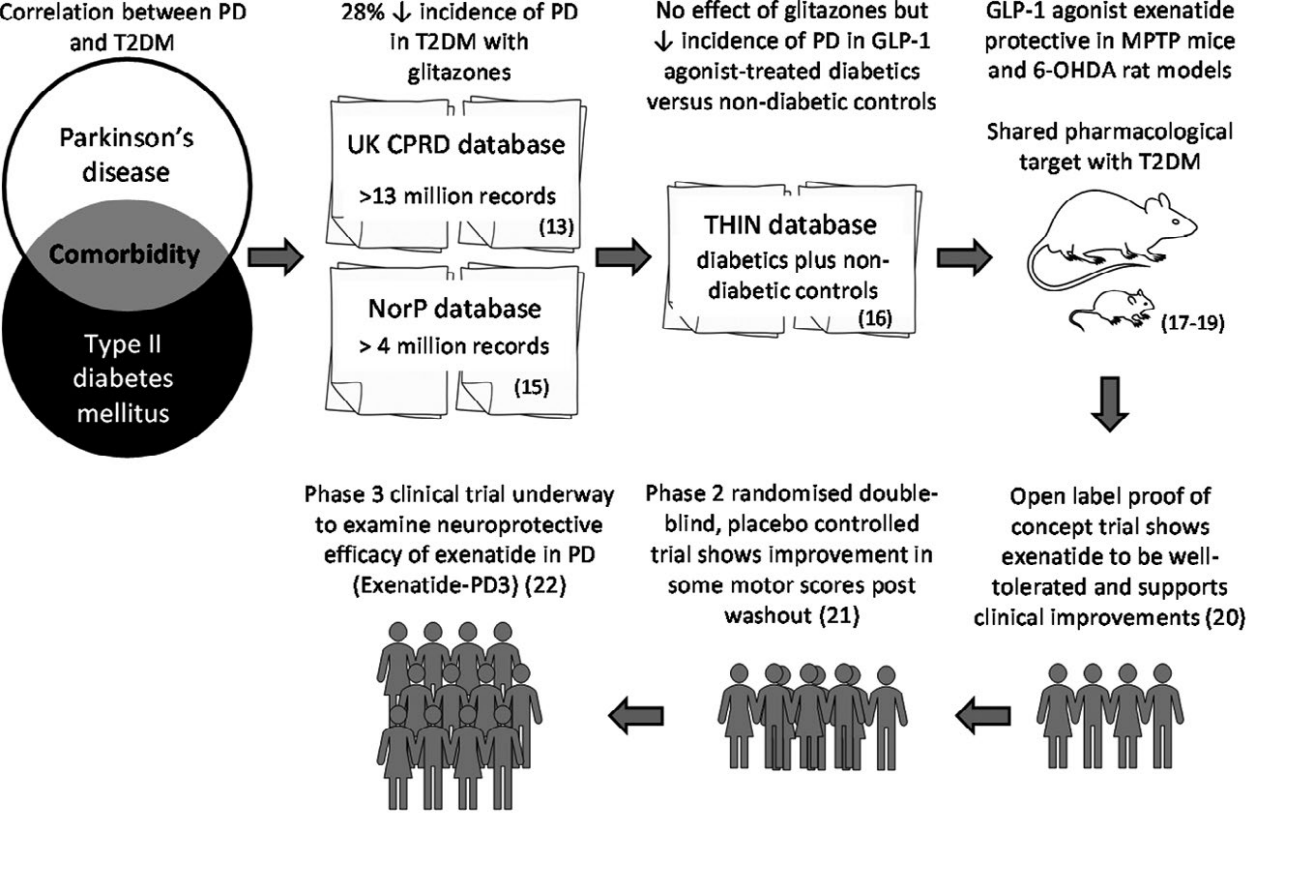
Schematic representation of the repurposing journey of the glucagon‐like‐peptide‐1 (GLP‐1) receptor agonist, exenatide in Parkinson's disease (PD). Type II diabetes mellitus (T2DM) was recognised as a potential risk factor for PD. Independent scrutiny of records from the UK Clinical Practice Research Datalink (CPRD) database and the Norwegian Prescription (NorP) database reported a consistent reduction in the incidence of PD in T2DM patients prescribed glitazones compared to those given treatments such as sulphonylureas or metformin. Subsequent interrogation of The Health Improvement Network (THIN) database revealed the incidence of newly diagnosed PD in T2DM patients receiving glitazones was not different to that in a matched non‐diabetic control group. However, those treated with GLP‐1 receptor agonists such as exenatide showed reduced incidence of PD. In pre‐clinical studies, exenatide provided neuroprotection in a GLP‐1 receptor‐dependent manner. Proof‐of concept and tolerability of exenatide was established via open label trial, with a follow‐up randomised placebo‐controlled trial revealing clinical improvement in some motor scores. Phase III multi‐centre clinical trials are now underway to assess the neuroprotective effects of exenatide in PD patients without co‐morbid T2DM (ClinicalTrials.gov Identifier: NCT04232969). Abbreviations: 6‐OHDA, 6‐hydroxydopamine; MPTP, 1‐methyl‐4‐phenyl, 1,2,3,6‐tetrahyropyridine. Numbers in parentheses indicate citations.

On the contrary, an excellent example where this strategy has been effective is the journey of exenatide (Figure 1). Type II diabetes mellitus (T2DM) has long been recognized as a potential risk factor for PD in most, but not all, epidemiological studies.^11^, ^12^ In 2015, Brauer et al. conducted a retrospective cohort study using the UK Clinical Practice Research Datalink database that contains primary care data on over 13 million people.^13^ They reported an approximate 28% reduction in the incidence of PD in T2DM patients prescribed glitazones when compared to those prescribed other antidiabetic treatments such as sulphonylureas or metformin.^13^ Interrogation of the Norwegian Prescription database (www.norpd.no)^14^ which hosts over 4 million Norwegian pharmacy records from 2004 onward corroborated this, reporting a similar 28% lower incidence rate of PD in T2DM patients prescribed glitazones compared to those prescribed metformin.^15^ However, subsequent scrutiny of The Health Improvement Network (THIN) database, containing data on over 100,000 people with T2DM, revealed that the incidence of newly diagnosed PD in T2DM patients receiving glitazones was in fact not different to that in a matched non‐diabetic control group over a median follow‐up of 3.33 years.^16^ In contrast, treatment with glucagon‐like peptide‐1 (GLP‐1) receptor agonists such as e xenatide, liraglutide, and lixisenatide was associated with a reduced incidence of PD in this same study. In preclinical studies, exenatide has been shown to be neuroprotective in both the 6‐hydroxydopamine (6‐OHDA)‐lesioned rat and the 1‐methyl‐4‐phenyl‐1,2,3,6‐tetrahydropyridine (MPTP)‐reated mouse models of PD.^17^, ^18^ This protection is lost when exenatide is co‐administered with a GLP‐1 receptor antagonist,^19^ confirming a shared pharmacological target mediating the efficacy of exenatide in both T2DM and PD. Importantly, these preclinical findings have so far shown good clinical translation. In the first proof of concept trial, Aviles‐Olmos et al. conducted an open‐label, single‐blind study with 45 individuals with moderate PD who either received s.c. exenatide (b.i.d.) for 12 months or were controls. Exenatide was mostly well tolerated and those receiving it showed a mean improvement compared to controls in motor and cognitive measures, which persisted after a 2‐month washout.^20^ A subsequent phase 2 trial with a double‐blind, randomized, placebo‐controlled design followed 60 participants receiving once‐weekly injections of long‐acting exenatide or placebo for 48 weeks, again with a 12‐week washout. Post washout, those in the exenatide group showed an improvement in OFF medication scores compared to the placebo group.^21^ On the basis of this collective evidence, a phase 3 clinical trial to assess the neuroprotective effects of exenatide in PD patients is now currently underway. This UK‐based multicenter double‐blind, randomized, placebo‐controlled trial will recruit 200 people with mild to moderate PD to examine the efficacy of long acting exenatide administered once‐weekly for 96‐weeks.^22^

The L‐type calcium Ca^2+^ channel blocker, israpdipine, typically prescribed as anti‐hypertensive medication is another good example of this approach. In a meta‐analysis of six observational cohort or case‐control studies, use of Ca^2+^ channel blockers was associated with a 19% reduction in PD risk compared to non‐users.^23^ Consistent with this, isradipine reduced cell death in preclinical rodent models of PD,^24^, ^25^ an effect thought to be driven at least in part by block of the pacemaking Ca _V_ 1.3 L‐type calcium channels that render nigrostriatal neurones particularly sensitive to degenerative insults.^26^ Despite these promising outcomes in preclinical studies, the recently completed phase 3 trial, STEADY‐PD III, found no slowing of clinical progression in early‐stage PD patients following 3 years’ treatment with isradipine.^27^ While disappointing, this failure serves to remind us that drugs identified through repurposing strategies, while likely to encounter reduced failure rates, are still not guaranteed to succeed in phase 3 trials.

While use of these healthcare‐centered databases has enabled research groups to correlate medication histories to disease prevalence on a substantial scale and thereby identify potential drugs for repurposing in PD, they have also been useful in following up on basic experimental observations. By way of example, the β _2_ ‐adrenoceptor agonist, salbutamol, was recently identified by high‐throughput gene expression assay as one of a number of drugs that reduced endogenous alpha synuclein gene expression in human neuroblastoma cells.^28^ This prompted Mittal et al. to interrogate the Norwegian Prescription database, thereby revealing an intriguing negative correlation between use of salbutamol and PD incidence, with a threefold reduction noted in users over a 5‐year period.^28^ These findings have since been supported by some subsequent large cohort based studies^29^ but refuted by others,^30^ so this association remains equivocal. Nevertheless, we have demonstrated the protective effects of salbutamol in the 6‐OHDA‐lesioned rat model of PD.^31^ In our studies, enhanced production of the endogenous growth factor, fibroblast growth factor 20 (FGF20), was identified as an additional potential protective mechanism of salbutamol. Whether these findings are translated through to clinical trials with salbutamol remains to be seen.

### 2.3 Novel target‐driven approaches

Another direct route to repurpose drugs for PD is based on the emergence of novel targets for which there are existing approved drugs. Novel targets are hypothesized based on susceptibility loci coinciding with genes of plausible functional significance in the disease. For example, both hereditary and sporadic forms of PD are often associated with the reduced activity of the E3 ubiquitin ligase, parkin.^32^ The risk associated with this reduced activity is postulated to be caused by compromised autophagy and reduced clearance of reactive oxygen species that are damaging to neurones.^33^ More recently, it has been found that abelson tyrosine protein kinase 1 (ABL1) activity is increased in the striatum and substantia nigra where it serves to inactivate parkin.^34^ Following identification of ABL1 as a target for PD treatment, nilotinib, an ABL inhibitor currently prescribed for leukemia, was taken into trials in the hope that ABL inhibition would prove beneficial through restored parkin activity. The phase 2 trials with nilotinib in PD have recently concluded that while being “reasonably safe” and demonstrating “acceptable tolerability” there was no clinical efficacy, and hints at worsening of motor function have understandably reduced enthusiasm for subsequent phase 3 trials.^35^, ^36^

A more promising example of this target‐driven process is ambroxol. Mutations in the GBA gene encoding glucocerebrosidase (GCase) are a major genetic risk factor for PD.^37^ The mutations result in reduced lysosomal GCase activity, a feature also evident in sporadic PD brain.^38^ The resultant impairment of lysosomal‐autophagy processes is believed to contribute to alpha‐synuclein build up. In 2009, screening of over 1000 FDA‐approved drugs for GCAse activity revealed ambroxol, a drug used since the 1970s as a cough linctus, as a positive hit.^39^ Increased GCase activity and reduced phospho‐alpha synuclein levels were subsequently noted following ambroxol treatment in cell‐based and mouse models of PD.^40^, ^41^ Results of the recently completed single center, open‐label, non‐controlled phase 2 clinical trial support ambroxol's further progression. This AiM‐PD trial found ambroxol to be safe and well tolerated and to produce an increase in alpha synuclein in the CSF,^42^ suggestive of brain clearance. Phase 3, placebo‐controlled trials are now being planned to establish whether ambroxol possesses disease‐modifying efficacy.

### 2.4 Targeted gene expression

Repurposing can take advantage of the downstream effects of drug activity in, for example, the perturbation of gene expression. One such project sourced compounds that elevate the expression of the glutamate transporter‐1, with the aim of attenuating neuronal loss from excitotoxicity seen in amyotropic lateral sclerosis.^43^ A reporter screen led to the identification of beta‐lactome antibiotics as potential therapeutics.^44^ Although expanded on below in the transcriptional profiling section, it is pertinent to note here that in silico screening of published transcription profile databases of FDA‐approved drugs, such as the connectivity mapping (CMAP) dataset^45^ also identified beta‐lactome antibiotics as drugs that could up‐regulate glutamate transporter‐1. This somewhat hybrid approach, harnessing the power of transcriptional databases to identify drugs to perturb a given target, allows the same endpoint to be achieved (in this case identifying drugs to elevate glutamate transporter‐1) with significantly lower costs and reduced time. At least one of the compounds identified from this screen, ceftriaxone, progressed into clinical trials for ALS, supporting this approach. Although showing positive outcomes in phase 1 and phase 2 stages,^46^ ceftriaxone failed, however, to reduce the decline in function, or increase lifespan in ALS patients.^47^

Growth factors are an obvious target for intervention in neurodegenerative disorders,^48^ and FGF20 had previously attracted our attention for its role in PD.^49^, ^50^ Adopting this same hybrid approach, we queried the CMAP dataset for drugs that could increase FGF20 transcription in order to identify prospective PD therapies.^31^ A significant fraction of the in silico‐derived candidates were shown to drive FGF20 protein expression in the substantia nigra or striatum of rats post‐oral administration. Two of these drugs, salbutamol (discussed earlier in relation to the observational studies) and triflusal showed protection in the 6‐OHDA‐lesioned rat model of PD. This in vivo exploration of drugs identified from in silico screening is attractive as it simultaneously answers the complementary questions of blood–brain barrier penetrance, action at the relevant target tissue (in this case the nigrostriatal pathway) and effectiveness at modulating production of the desired target protein (in this case FGF20). In support of this approach, the identification of salbutamol as a potential neuroprotective drug is consistent with the previously described epidemiological findings of a reduced risk of developing PD in salbutamol users.^28^, ^29^

### 2.5 Transcriptional profiling

Transcription‐based drug repurposing is rooted in the observation that disease‐associated gene expression profiles are consistent and can thereby serve as quantitative disease phenotypes.^51^, ^52^ The hypothesis is that drugs tending to drive gene expression in a reverse sense to that seen in the disease state may ameliorate the condition.^53^, ^54^ This approach has resulted in many successful projects^55^, ^56^, ^57^, ^58^, ^59^, ^60^, ^61^ with some drugs that showed excellent efficacy in animal models, such as topiramate for inflammatory bowel disorder, being subsequently examined for clinical benefits, albeit with less successful outcomes.^62^

Nevertheless, this methodology is likely applicable to neurodegenerative conditions based on the observation that both Alzheimer's disease (AD) and PD have characteristic expression profiles and that drugs with reverse profiles to AD have already been shown to be enriched for neuroprotective activities.^63^, ^64^

Transcription‐based repurposing is facilitated by the extensive and publicly available drug‐associated transcription data that can be interrogated with user‐friendly web tools, such as SPIED.^60^ In particular, the aforementioned CMAP dataset^45^ comprises the profiles of 1309 drug‐like compounds profiled on cancer cell lines. The Library of Integrated Network‐Based Cellular Signatures^65^ iteration of CMAP has greatly expanded the number of compounds to over 15,000 and crucially includes profiles generated on over 100 induced pluripotent stem cell (iPSC) derived cell lines, including cortical neurones. This data inflation has come at the price of basing the profiles through a linear model fit on the measured expression of just 1000 landmark genes.

In a recent study on transcription‐based repurposing in AD, the full transcriptional profiles of 153 drugs that were long list AD repurposing candidates, based on a CMAP analysis, were fully profiled on iPSC‐derived cortical neuronal cultures. Seventy‐eight of the drugs were shown to maintain their anticorrelation with expression changes in AD.^64^ Furthermore, 19 of the 78 iPSC‐thresholded drugs showed significant reversal of AD‐associated changes in at least two independent assays,^66^ adding weight to this approach. However, it should be noted that as well as comprising a deleterious component, the disease‐associated transcriptional profile will in general harbor a defensive—or positive compensatory—component and thus reversing the whole profile may not always be beneficial. For example, in a repurposing project directed to the COVID‐19 pandemic it was found that severe acute respiratory syndrome infection induces lung cells to elevate viral defense pathways and so drugs recapitulating these expression changes tend to harbor antiviral activity.^67^ It is therefore important to disentangle the gene expression changes that are protective from those that are pathological, which is not a trivial task. We are currently in the process of doing just this for PD disease‐associated profiles.

One obvious method would be to determine which gene sets are enriched in PD and use expert knowledge to decide which of these to target and how their expression should be modulated. An example of a more complex approach would be the network‐based method used by Chandran et al. to discover drugs that promote regeneration in rodent spinal cord injury models.^68^ It is known that the peripheral nervous system has good regenerative capacity following injury, while the central nervous system does not. The disparity in this capacity is often ascribed to differences in transcriptional responses between the tissues. Using Weighted Gene Co‐expression Network Analysis, Chandran et al. attempted to identify groups of genes expressed in the regenerating peripheral nervous system but not in the central nervous system and then pharmacologically induce their expression in the spinal cord to aid regeneration. This approach showed some promise as ambroxol was identified and shown to enhance axonal regeneration in vivo.^68^

Transcription‐based repurposing in its simplest incarnation directly compares the transcription profiles of the disease state with that of the compound. However, it may be of value when identifying drug targets to integrate other sources of information into the selection process. To this end, Gao et al. developed a scoring system that ranked genes based on (i) their fold‐change in the disease state (in this case PD), (ii) how related each gene was to other differentially expressed genes in that network and (iii) the number of “therapeutic” molecules associated with each PD‐related gene in the Comparative Toxicogenomics Database (https://ctdbase.org/).^69^ The top 20 ranked genes were used to query CMAP, and 11 therapeutic small molecules were identified, of which the heat shock protein 90 inhibitor, 17‐dimethylaminoethylamino‐17‐demethoxygeldanamycin (17‐DMAG, or alvespimycin), was selected for further investigation. 17‐DMAG was shown to protect against rotenone toxicity in SHSY‐5Y neuroblastoma cells.^70^ Gao et al. claim that selecting the top 20 ranked genes resulted in better query performance on the CMAP database than using all differentially expressed genes, as measured by the enrichment factor metric and the number of therapeutic molecules identified. While other studies have now shown 17‐DMAG to attenuate neuropathology and motor impairment in a mouse model of spinocerbellar ataxia type 3,^71^ no follow‐up study has yet occurred in animal models of PD to lend weight to this particular scoring system.

High content phenotypic data associated with drug action can be harnessed to discover alternative candidate therapeutics with more favorable pharmacology. Global transcription profiling provides an effective quantitative phenotype allowing for the direct comparison of compounds.^45^ Another methodology is provided by the National Cancer Institute's Compare portal^72^ mapping compound action to the growth inhibitory data profile of over 59 cancer cell lines for nearly 100,000 drugs. This resource has been applied to the current coronavirus COVID‐19 pandemic and shown to link disparate, chemically unrelated drugs with reported antiviral activities.^73^ Such approaches allow compounds to be repurposed without reference to the inhibition or agonism of specific targets but instead based on their ability to alter the abundance of target proteins, in a similar manner to that adopted in our FGF20 study.^31^

### 2.6 Genome‐informed target identification

Genome wide association studies (GWAS) attempt to identify genetic variants in the population that are correlated with an increased risk of PD. For the goal of drug repurposing this method can be utilized to narrow down the list of genes and molecular pathways associated with PD risk loci, so that druggable targets can be identified for further investigation. GWAS data are widely available for meta‐analysis. In a recent application to PD, Uenaka et al.^74^ collated a list of 32 risk genes from the GWAS literature and expanded this to a set of 866 protein targets based on direct interactions reported in protein‐protein interaction (PPI) databases (InWeb and PINA) of which 48 were targets of 57 FDA approved drugs. Five drugs from this long list with previously reported neuroprotective activities were selected for further testing (dasatinib, duloxetine, furosemide, regorafenib, and dabrafenib). Of these, dabrafenib, a drug approved for melanoma, provided the most robust increase in cell viability in vitro. Furthermore, it was shown to prevent dopaminergic neuronal loss in the MPTP‐treated mouse model of PD, when administered 3 days prior to MPTP exposure^74^ although, no studies appear to have yet followed up on this observation.

Exploiting the concept of genetic pleiotropy may additionally yield some interesting drug candidates for repurposing between diseases. Genetic pleiotropy refers to the involvement of one gene in multiple phenotypes. By extension of this concept, mutations in a single‐gene could increase the risk of multiple seemingly unrelated diseases. In the case of PD, Pickrell et al.^75^ reported that a nonsynonymous variant in zinc transporter 8, SLC39A8 is associated with the risk of schizophrenia and PD. Furthermore, genetic links have been found between PD and many autoimmune diseases, including type 1 diabetes, Crohn's disease, ulcerative colitis, rheumatoid arthritis, celiac disease, psoriasis, and multiple sclerosis.^76^ This further supports the debated hypothesis that immune‐modulating drugs have potential as therapeutics for PD. Epidemiological and observational studies report a 15%–17% reduced risk of developing PD with non‐aspirin‐based NSAIDs,^77^, ^78^ while a more recent meta‐analysis found no evidence for a reduced risk.^79^

In the likely event of high‐risk alleles not emerging from GWAS, researchers must deal with large numbers of moderate risk loci. Variants are then prioritized based on whether they have a direct effect on genes or are located in regions of genomic function. Gene pathways can be scored against collections of risk alleles to power statistical conclusions that can inform on the biology underlying the disease. One approach to getting a direct biological readout for genetic variation is through large‐scale transcription profiling of tissue samples from genotyped donors. A database of these expression qualitative trait loci (eQTL) is hosted by the Broad Institute Genotype‐Tissue Expression (GTEx) portal.^80^ Methods have been developed to map GWAS data onto global gene expression patterns based on training models on the GTEx datasets.^81^ In the absence of individualized variant data, researchers must make do with GWAS summary data and it has been claimed that meaningful gene expression patterns can be extracted from this data,^82^ though it is not clear to what extent these expression patterns are shared across the disease cohort in the original GWAS study. However, the technique has recently been applied to GWAS data from various psychiatric conditions.^83^ Here, GWAS summary data were mapped onto disease associated expression patterns that were queried against the CMAP database of compound driven gene expression patterns. Interestingly, in support of this approach, the AD profile derived compounds showed a notable enrichment for NSAIDs in the candidate list.

## 3 FINAL THOUGHTS

The formidable challenges thrown up by complex multifactorial neurodegenerative conditions such as PD have led researchers to investigate alternatives to traditional target based novel entity pipelines. Drug repurposing draws its potential from the increasingly detailed and extensive data describing both disease states and drug activities. As such this form of drug discovery is open to the academic laboratory where therapeutic potential can be firmed up through in vitro and in vivo experimentation. Drugs with therapeutic potential can then be rapidly progressed to the clinic based on their established safety profiles and prescription history. In this review, we have surveyed the various repurposing methodologies, focusing on those that have been applied to PD and those with potential applicability. In particular, we have highlighted from our own previous work a technique whereby potential therapeutic compounds can emerge on the basis of their stimulating brain cells to produce the FGF20 growth factor with the aim of slowing neurodegeneration in PD. This approach circumvents the difficulties faced in delivering growth factors to the brain as encountered in the recent glial‐derived neurotrophic factor trial in PD.^84^ We have also discussed the potential of global transcriptomics to inform drug discovery, highlighting work in AD and the potential application to PD, with some more speculative approaches introduced to illuminate how the future landscape in drug repurposing in PD may look. Most of the above drug repurposing strategies are still in their infancy and time will tell how effective they really are at introducing new drugs into the clinic for complex neurodegenerative conditions like PD.

## NOMENCLATURE OF TARGETS AND LIGANDS

Key protein targets and ligands in this article are hyperlinked to corresponding entries in http://www.guidetopharmacology.org, the common portal for data from the IUPHAR/BPS Guide to PHARMACOLOGY^85^ and are permanently archived in the Concise Guide to PHARMACOLOGY 2019/20.^86^

## DISCLOSURE

The authors (EJRF, TK, GW, and SD) have no financial or non‐financial conflicts of interests in relation to the content of this review.

## Data Availability

There is no original data contained within this review. All original sources are referenced.
